# Strain Ventricular em uma População Adulta Brasileira do Estudo ELSA-Brasil: Valores de Referência e seus Determinantes

**DOI:** 10.36660/abc.20240634

**Published:** 2025-05-30

**Authors:** Eduardo Gatti Pianca, Murilo Foppa, Giulia Bevilacqua Schmitz, Wilson Cañon-Montañez, Bruce Bartholow Duncan, Angela Barreto Santiago Santos

**Affiliations:** 1 Universidade Federal do Rio Grande do Sul Programa de Pós-Graduação em Cardiologia e Ciências Cardiovasculares Faculdade de Medicina Porto Alegre RS Brasil Universidade Federal do Rio Grande do Sul – Programa de Pós-Graduação em Cardiologia e Ciências Cardiovasculares, Faculdade de Medicina, Porto Alegre, RS – Brasil; 2 Hospital de Clinicas de Porto Alegre Porto Alegre RS Brasil Hospital de Clinicas de Porto Alegre, Porto Alegre, RS – Brasil; 3 Universidad de Antioquia Faculty of Nursing Medellin Colômbia Universidad de Antioquia – Faculty of Nursing, Medellin – Colômbia

**Keywords:** Deformação Longitudinal Global, Ventrículos do Coração, Estudos de Coortes, Ecocardiografia, Função Ventricular

## Abstract

**Fundamento:**

O comprometimento da função do ventrículo esquerdo (VE) e do ventrículo direito é um importante preditor de risco cardiovascular. O strain longitudinal global (SLG) fornece sensibilidade superior para avaliar a função sistólica em comparação aos parâmetros tradicionais, aumentando a precisão diagnóstica em várias condições cardíacas. No entanto, faltam valores de referência para SLG em diversas populações.

**Objetivos:**

Estabelecer valores de referência para SLGVE e strain longitudinal da parede livre do ventrículo direito (SLPLVD) em uma população multiétnica brasileira sem fatores de risco cardiovascular ou doença. Também exploramos como fatores clínicos e ecocardiográficos influenciam a distribuição do SLG, abordando uma lacuna nas diretrizes globais que geralmente dependem de dados de populações homogêneas ou geograficamente distantes.

**Métodos:**

Incluímos 1.048 participantes da coorte ELSA-Brasil que foram submetidos à ecocardiografia com análise do SLG. Uma subamostra saudável (n = 527) foi definida pela exclusão de indivíduos com doença cardiovascular ou renal, hipertensão ou diabetes para estabelecer intervalos de referência do SLG. A prevalência de SLG anormal foi avaliada, e foram identificados fatores associados à redução de SLGVE e SLPLVD. A significância estatística foi definida como p < 0,05.

**Resultados:**

Na subamostra saudável (idade média de 50,2 anos, 59% do sexo feminino), a média de SLGVE foi de 19,0% (intervalo de confiança de 95%: 14,3 a 23,8), e a média de SLPLVD foi de 28,3% (intervalo de confiança de 95%: 22,3 a 34,3). As mulheres exibiram valores mais altos de SLGVE e SLPLVD do que os homens, sem diferenças significativas relacionadas à idade. Valores anormais de SLGVE e SLPLVD foram observados em 3,8% e 1,6% dos participantes, respectivamente. O SLGVE mais baixo foi associado à obesidade, hipertensão e diabetes; o SLPLVD reduzido foi correlacionado com maior índice de massa corporal e massa do VE.

**Conclusões:**

Propomos valores de referência para SLGVE e SLPLVD em uma grande coorte brasileira, destacando associações com comorbidades cardiovasculares e estrutura ventricular.

## Graphical abstract

**Figura Central:**
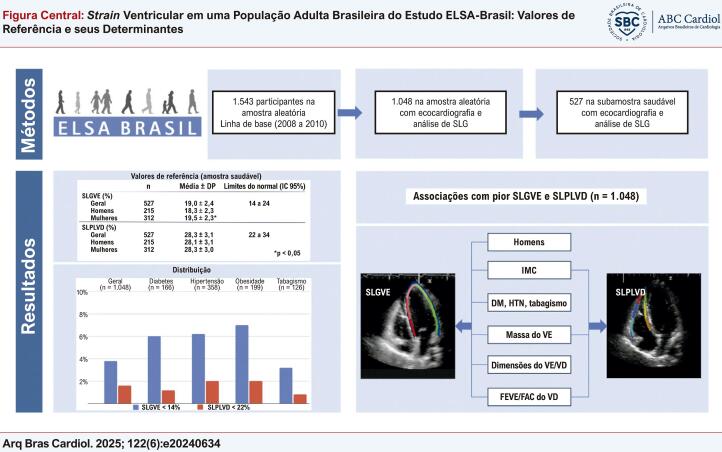
Strain Ventricular em uma População Adulta Brasileira do Estudo ELSA-Brasil: Valores de Referência e seus Determinantes DM: diabetes mellitus; DP: desvio padrão; FAC: variação da área fracionada; FEVE: fração de ejeção do ventrículo esquerdo; HTN: hipertensão; IC: intervalo de confiança; IMC: índice de massa corporal; SLG: strain longitudinal global; SLPLVD: strain longitudinal da parede livre do ventrículo direito; VD: ventrículo direito; VE: ventrículo esquerdo.

## Introdução

O comprometimento da função sistólica do ventrículo esquerdo (VE) é um preditor bem conhecido de morbimortalidade cardiovascular,^1^ e é geralmente relatado e categorizado por meio da fração de ejeção ventricular esquerda (FEVE). No entanto, a FEVE tem limitações reconhecidas em relação à reprodutibilidade e padronização, diminuindo a detecção de anormalidades sutis na função do VE.^2^ A avaliação da contratilidade do VE por meio do strain miocárdico usando speckle tracking superou a maioria dessas restrições. Ademais, essa técnica é resiliente a variações nos planos de aquisição e na direção do movimento muscular, permitindo assim a avaliação com imagens ecocardiográficas bidimensionais regulares.^3^ Particularmente, a análise do strain longitudinal global (SLG) demonstrou ser uma medida robusta para detectar disfunção sistólica precoce e um parâmetro prognóstico além da FEVE.^4,5^

A insuficiência do ventrículo direito (VD) também está emergindo como um parâmetro prognóstico em cenários como insuficiência cardíaca, infarto do miocárdio e hipertensão pulmonar.^6^ No entanto, a avaliação da função do VD por meio da ecocardiografia convencional, como a velocidade sistólica máxima do anel lateral tricúspide por Doppler tecidual pulsátil (s’) e a excursão sistólica do plano anular tricúspide (TAPSE), é limitada devido ao formato complexo dessa câmara e à avaliação restrita aos segmentos basais em vez de todo o VD. Para enfrentar esse desafio, parâmetros emergentes como o strain do VD provaram ser confiáveis e precisos, com a vantagem de serem menos afetados pela dependência do ângulo do que os parâmetros tradicionais de função.^6^ Além disso, o strain longitudinal do VD aumentou a precisão diagnóstica em várias condições cardíacas, incluindo hipertensão pulmonar, embolia pulmonar e displasia arritmogênica do VD.^6^ Também auxilia na estratificação adicional do prognóstico entre pacientes com insuficiência cardíaca, síndromes coronárias agudas e transplante cardíaco.^6^

Apesar de seus pontos fortes, as medições do SLGVE e do strain longitudinal da parede livre do ventrículo direito (SLPLVD) apresentam várias limitações que devem ser consideradas ao interpretar os resultados na prática clínica e na pesquisa. Essas limitações decorrem principalmente de questões relacionadas à qualidade da imagem (como posição corporal do paciente, janelas acústicas desfavoráveis ou artefatos de movimento, todos os quais podem prejudicar significativamente a análise de deformação), variabilidade da frequência cardíaca (onde frequências cardíacas muito altas ou muito baixas podem afetar o tempo de contração miocárdica e medição de strain, potencialmente distorcendo os resultados) e variabilidade de software (já que diferentes plataformas de softwares comerciais podem empregar algoritmos ligeiramente diferentes para cálculo de strain, levando à variabilidade nas medições do SLG). Além disso, o SLPLVD é inerentemente mais desafiador de avaliar devido à anatomia e função complexas do RV. Entender essas limitações é essencial para a interpretação precisa das medidas de strain e para evitar a interpretação excessiva dos resultados, particularmente em pacientes com anormalidades limítrofes ou sutis.^7^

A Sociedade Americana de Ecocardiografia e a Associação Europeia de Ecocardiografia estabeleceram valores de referência padrão para medidas de ecocardiografia.^8^ No entanto, esses dados podem não representar com precisão populações mundiais diversas, como já demonstrado em estudos anteriores que incluíram a FEVE.^9^ Embora as populações globais possam servir como ponto de partida, as variações genéticas, sociais e ambientais entre países e continentes sugerem que os valores de referência universais podem ser imprecisos para o diagnóstico clínico. A necessidade de valores de referência locais se torna ainda mais relevante em países com populações multiétnicas, como o Brasil, onde as características genéticas e comportamentais podem variar consideravelmente entre diversos grupos étnicos. Para garantir uma avaliação mais precisa da função ventricular em pacientes dessa população, é crucial definir valores de referência específicos para o Brasil, com base nas características da população local. O Estudo ELSA-Brasil apresenta uma oportunidade única de estabelecer limites de referência para parâmetros de SLG de uma grande amostra de adultos brasileiros, permitindo melhor interpretação dos resultados da ecocardiografia em nossa população enquanto a implementação desses parâmetros ocorre na prática clínica diária. Com base em uma população multiétnica brasileira de meia-idade, visamos: (1) descrever valores de referência para SLGVE e SLPLVD em uma subamostra livre de doença cardiovascular e fatores de risco; (2) descrever a prevalência de SLG anormal nessa coorte ocupacional, usando o ponto de corte da subamostra saudável; (3) avaliar a influência de fatores clínicos e ecocardiográficos na distribuição de SLG dentro dessa coorte ocupacional.

## Métodos

### População do estudo

**Figura 1:**
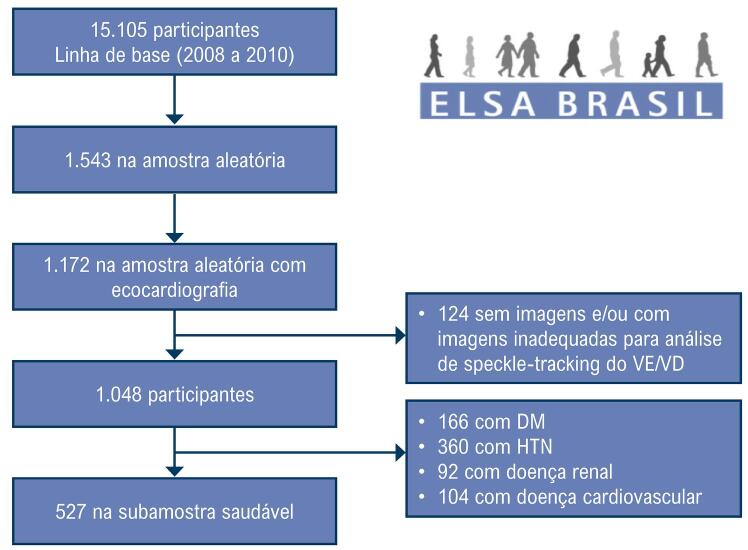
– Fluxograma do estudo. DM: diabetes mellitus; HTN: hipertensão; VD: ventrículo direito; VE: ventrículo esquerdo.

O Estudo Longitudinal de Saúde do Adulto (ELSA-Brasil) é um estudo epidemiológico prospectivo projetado para investigar doenças cardiovasculares e diabetes em 15.105 participantes, servidores públicos de universidades ou instituições de pesquisa em 6 cidades brasileiras (São Paulo, Rio de Janeiro, Belo Horizonte, Vitória, Salvador e Porto Alegre). Todos os funcionários ativos ou aposentados com idade entre 35 e 74 anos foram elegíveis para o estudo. Os detalhes do estudo, incluindo o desenho, os critérios de elegibilidade, as fontes e os métodos de recrutamento e as medidas obtidas foram descritos em publicações anteriores.10,11 A presente investigação foi um estudo transversal do ELSA-Brasil durante a primeira visita (de agosto de 2008 a dezembro de 2010), a partir de uma subamostra aleatória predefinida compreendendo 10% da amostra total. Dessa amostra, incluímos participantes que foram submetidos a um exame ecocardiográfico com análise adequada do VE e do VD e que estavam em ritmo sinusal durante o exame. A análise para a definição dos valores de referência de SLGVE e SLPLVD foi restrita a uma subamostra de participantes “saudáveis” composta por aqueles livres de hipertensão; diabetes; doença renal, definida como uma taxa de filtração glomerular < 60 mL/min/1,73 m^2^ ou relação albumina/creatinina urinária ≥ 30 mg/g; e condições cardiovasculares preexistentes, que foram definidas como histórico de acidente vascular cerebral, insuficiência cardíaca ou infarto do miocárdio (Figura 1).

O Estudo ELSA-Brasil foi aprovado pelos comitês de ética em pesquisa das instituições envolvidas, e todos os participantes forneceram consentimento informado por escrito.

### Ecocardiografia

Todos os ecocardiogramas transtorácicos foram obtidos na primeira visita do estudo em 6 centros diferentes. As imagens foram adquiridas em máquinas de ecocardiografia configuradas de forma idêntica (Aplio XG, Toshiba) com um transdutor de setor de 2,5 MHz, e a taxa de quadros para a aquisição variou de 40 a 60 quadros por segundo, de acordo com protocolos padronizados. Esses protocolos incluíram as janelas paraesternais do eixo longo e curto, bem como as janelas apicais de 4 câmaras e 2 câmaras. Os exames foram gravados em formato digital e transferidos para o centro de leitura ecocardiográfica do ELSA-Brasil em Porto Alegre, Rio Grande do Sul. Os parâmetros ecocardiográficos padrão e Doppler foram analisados usando uma estação de trabalho offline (ComPACS 10.5 workstation; Medimatic SrL, Itália). Todas as medições foram feitas em triplicado seguindo as recomendações da Sociedade Americana de Ecocardiografia,8 incluindo diâmetros do VE, espessura da parede do VE, massa do VE, FEVE, volume do átrio esquerdo (AE), velocidades de influxo mitral diastólico precoce e tardio, velocidades do anel mitral, diâmetro do VD, áreas diastólica e sistólica do VD e variação da área fracionada (FAC, sigla do inglês fractional area change) do VD.

Para avaliar os parâmetros da disfunção sistólica precoce, seguimos as diretrizes e recomendações atuais.12 A função da deformação miocárdica do VE e VD foi medida usando um software previamente validado e comercialmente disponível dedicado à análise do VE e VD (2D Cardiac Performance Analysis©, TomTec-ArenaTM 1.2 Imaging Systems, Unterschleißheim, Alemanha). Para avaliação do strain do VE, as bordas endocárdicas foram traçadas no quadro diastólico final de imagens bidimensionais adquiridas das janelas apicais de 2 e 4 câmaras. A diástole final foi definida pelo complexo QRS, ou como o quadro após o fechamento da válvula mitral. A sístole final foi definida pela visualização do quadro antes da abertura da válvula mitral na janela paraesternal do eixo longo. Speckle tracking foi realizado quadro a quadro dentro do miocárdio do VE ao longo de 1 ciclo cardíaco; subsequentemente, regiões de interesse basal, média e apical foram criadas. Cada imagem foi cuidadosamente inspecionada e os segmentos em que houve falha no rastreamento foram ajustados manualmente. Se mais de 1 segmento não pudesse ser rastreado, ou se houvesse uma falta de um ciclo cardíaco completo ou encurtamento significativo do VE, as medições eram consideradas não confiáveis, e o paciente era excluído da análise. O SLGVE foi calculado como o strain longitudinal médio nas janelas apicais de 2 e 4 câmaras. Para a avaliação do strain longitudinal do VD, a diástole final foi definida manualmente pelo complexo QRS, enquanto a sístole final foi definida como uma abertura da válvula tricúspide na janela de 4 câmaras. A parede livre do VD e o septo interventricular foram divididos em 3 segmentos (apical, médio e basal). O SLPLVD é o valor médio dos 3 segmentos da parede livre do VD. Todos os valores de SLG são apresentados como valores absolutos e foram obtidos durante a fase de pico de contração do ciclo cardíaco (pico de strain sistólico).

A reprodutibilidade intra e interobservador para as medidas de SLGVE e SLPLVD foi previamente avaliada em 50 casos selecionados aleatoriamente da coorte ELSA-Brasil. Para SLGVE, os coeficientes de variação intra e interobservador foram de 5,4% e 7,4%, com coeficientes de correlação intraclasse de 0,83 (intervalo de confiança [IC] de 95%: 0,73 a 0,90) e 0,76 (IC 95%: 0,61 a 0,86), respectivamente.13 Para SLPLVD, os coeficientes de variação foram de 5,1% e 8,3%, com valores de coeficiente de correlação intraclasse de 0,78 (IC de 95%: 0,67 a 0,89) e 0,54 (IC de 95%: 0,34 a 0,74), respectivamente.^14^

### Análises estatísticas

Os dados contínuos com distribuição normal foram apresentados como média e desvio padrão. Os dados contínuos com distribuição anormal foram exibidos como mediana e intervalo interquartil, e os dados categóricos foram mostrados como total e proporção. A normalidade foi avaliada usando o teste de Shapiro-Wilk. Variáveis contínuas foram comparadas usando o teste t bilateral com variância desigual para dados distribuídos normalmente, o teste de postos sinalizados de Wilcoxon para dados distribuídos anormalmente e o teste qui-quadrado para variáveis categóricas. Apresentamos os valores médios e o IC de 95% correspondente para SLGVE e SLPLVD, definindo valores absolutos abaixo desse ponto de corte na amostra saudável como anormais. Categorizamos a amostra em tercis de acordo com a gravidade da função sistólica do VE e do VD medida por SLG, e aplicamos testes de tendência (teste t bilateral com variância desigual, teste de postos sinalizados de Wilcoxon e teste qui-quadrado) para ilustrar a associação de SLG com características demográficas e medidas ecocardiográficas de estrutura e função cardíaca. A análise de regressão linear foi realizada para avaliar a associação entre FEVE e SLGVE, bem como entre SLPLVD e FAC do VD.

Todas as análises foram realizadas com o pacote de software STATA (versão 13, Stata Corp, College Station, Texas, Estados Unidos). Todos os testes foram bilaterais, e foram considerados estatisticamente significativos valores de p < 0,05.

## Resultados

Nossa população total (n = 1.048) tinha uma idade média de 52 anos e incluía 559 (53%) mulheres. O maior centro de recrutamento foi São Paulo (26,7%), seguido por Belo Horizonte (22,6%), Porto Alegre (16,6%), Salvador (13,7%), Rio de Janeiro (13,1%) e Vitória (7,1%). A Tabela 1 apresenta as características clínicas da população de estudo estratificadas por sexo. A idade não diferiu entre os sexos; entretanto, os indivíduos do sexo masculino apresentaram maior área de superfície corporal, prevalência de hipertensão, diabetes e tabagismo atual, bem como piores escores de risco cardiovascular. Em relação aos parâmetros ecocardiográficos, as mulheres apresentaram cavidades menores (diâmetros do AE, VE e VD), menor massa do VE e espessura relativa da parede, além de maior razão E/e’ em comparação aos homens. Os parâmetros de função sistólica e deformação miocárdica para o VE e VD foram maiores nas mulheres do que nos homens, incluindo maior FEVE, SLGVE, FAC do VD e SLPLVD (Tabela 2).

**Tabela 1:** – Características clínicas basais da população do estudo

	Geral (n = 1.048)	Homens (n = 489)	Mulheres (n = 559)	Valor p
**Idade, anos**	52 ± 8,7	51,9 ± 9	52,1 ± 8,4	0,77
**Raça autodeclarada, n (%)**				
Branca	523 (49,9)	248 (49,2)	277 (50,4)	0,7
Preta	184 (17,5)	79 (16,1)	105 (18,7)	0,26
Parda	293 (27,9)	145 (29,6)	148 (26,4)	0,25
Indígena	13 (1,2)	8 (1,6)	5 (0,9)	0,27
Asiática	28 (2,7)	13 (2,6)	15 (2,7)	0,98
**Altura, cm**	165,3 ± 9,2	172,1 ± 7,2	159,4 ± 6,2	<0,01
**ASC, m^2^**	1,8 ± 0,2	1,92 ± 0,17	1,59 ± 0,15	<0,01
**IMC, kg/m^2^**	26,6 ± 4,3	26,7 ± 4,1	26,5 ± 4,5	0,43
**Hipertensão, n (%)**	358 (34,1)	191 (39)	167 (29,8)	<0,01
**Diabetes, n (%)**	166 (15,8)	100 (20,4)	66 (11,8)	<0,01
**Tabagismo atual, n (%)**	126 (12)	63 (12,8)	63 (11,2)	<0,01
**Obesidade, n (%)**	199 (18,9)	86 (17,5)	113 (20,2)	0,27
**Sobrepeso, n (%)**	446 (42)	231 (47,2)	215 (38,4)	<0,01
**Insuficiência cardíaca, n (%)**	14 (1,3)	6 (1,2)	8 (1,4)	0,78
**Infarto do miocárdio prévio, n (%)**	20 (3,6)	12 (1,1)	8 (1,6)	0,22
**Doença renal crônica, n (%)**	92 (8,7)	56 (11,4)	36 (6,4)	0,13
**Doença pulmonar obstrutiva crônica, n (%)**	15 (1,4)	4 (0,8)	11 (1,9)	0,11
**Acidente vascular cerebral, n (%)**	10 (0,9)	3 (0,6)	7 (1,2)	0,28
**Pontuação de risco de DCVA (%)**	2,7 [1,1 a 6,8]	5,3 [2,4 a 11]	1,4 [0,7 a 3,7]	<0,01

Os números representam média ± desvio padrão para variáveis contínuas com distribuição normal, mediana [intervalo interquartil] para variáveis contínuas com distribuição anormal e n (%) para variáveis categóricas. ASC: área de superfície corporal; DCVA: doença cardiovascular aterosclerótica; IMC: índice de massa corporal.

**Tabela 2:** – Parâmetros ecocardiográficos da população do estudo

	Geral (n = 1.048)	Homens (n = 489)	Mulheres (n = 559)	Valor p
**Diâmetro do AE, cm**	3,5 ± 0,4	3,7 ± 0,4	3,4 ± 0,4	<0,01
**Volume do AE, mL**	48 ± 13,6	51,1 ± 14,6	45,5 ± 12,2	<0,01
**Volume do AE indexado à ASC, mL/m2**	26,7 ± 6,7	26,5 ± 6,9	26,8 ± 6,5	0,61
**Diâmetro diastólico do VE, cm**	4,5 ± 0,4	4,7 ± 0,4	4,3 ± 0,4	<0,01
**Diâmetro sistólico do VE, cm**	2,8 ± 0,4	3 ± 0,4	2,7 ± 0,3	<0,01
**Volume diastólico final do VE, mL**	91 ± 18,5	99,7 ± 18,2	83,8 ± 15,5	<0,01
**Volume diastólico final do VE indexado à ASC, mL/m2**	51,6 ± 10,7	53,9 ± 11,3	49,7 ± 9,7	<0,01
**Volume sistólico final do VE, mL**	30,8 ± 10,9	35,2 ± 12,3	27,3 ± 8,2	<0,01
**Volume sistólico final do VE indexado à ASC, mL/m2**	17,1 ± 5,6	18,4 ± 6,3	16,1 ± 4,7	<0,01
**Massa do VE indexada à ASC, g/m2**	74,9 ± 17	82,7 ± 17,3	69,2 ± 14,5	<0,01
**LV massa/altura2,7, g/m2,7**	34,7 ± 8,6	36,2 ± 8,9	33,4 ± 8,1	<0,01
**Padrões geométricos do VE, n (%)**				
Remodelamento concêntrico	306 (29,1)	141 (28,8)	165 (29,5)	0,8
Hipertrofia concêntrica	49 (4,7)	20 (4,1)	29 (5,2)	0,4
Hipertrofia excêntrica	48 (4,6)	18 (3,7)	30 (5,4)	0,19
**Espessura relativa da parede**	0,4 ± 0,068	0,41 ± 0,069	0,4 ± 0,068	0,03
**Relação E/e’ mitral**	7,3 ± 1,9	7 ± 1,9	7,5 ± 1,9	<0,01
**Média do anel mitral e’, cm/s**	10,1 ± 2,4	9,8 ± 2,3	10,3 ± 2,5	<0,01
**Diâmetro basal do VD, cm**	3,5 ± 0,4	3,7 ± 0,3	3,3 ± 0,3	<0,01
**FEVE (método de Simpson), %**	59,5 ± 6,3	55,6 ± 6,3	60,2 ± 6,2	<0,01
**SLGVE, %**	18,6 ± 2,5	17,9 ± 2,5	19,2 ± 2,4	<0,01
Branca (n = 523)	18,6 ± 2,5	17,9 ± 2,4	19,3 ± 2,4	<0,01
Preta (n = 184)	18,4 ± 2,6	17,7 ± 2,7	19 ± 2,4	<0,01
Parda (n = 293)	18,7 ± 2,6	18 ± 2,5	19,4 ± 2,4	<0,01
Indígena (n = 13)	18,7 ± 2,3	18,2 ± 2,8	19,5 ± 1,4	0,3
Asiática (n = 28)	18,3 ± 2,4	17,7 ± 2,7	18,9 ± 2,2	0,19
**FAC do VD, %**	43,8 ± 5,6	43,1 ± 5,7	44,4 ± 5,5	<0,01
**SLPLVD, %**	28,2 ± 3,1	28 ± 3,2	28,4 ± 3,1	0,03
Branca (n = 523)	28,3 ± 3,1	27,9 ± 3,1	28,6 ± 3,1	0,02
Preta (n = 184)	27,9 ± 3,4	28,1 ± 3,7	27,8 ± 3,2	0,6
Parda (n = 293)	28 ± 2,9	27,8 ± 2,9	28,2 ± 2,9	0,3
Indígena (n = 13)	29,5 ± 2,8	29,2 ± 3,1	29,9 ± 2,6	0,6
Asiática (n = 28)	28,5 ± 2,6	27,6 ± 2,5	29,2 ± 2,5	0,11

Os números representam média ± desvio padrão. AE: átrio esquerdo; ASC: área de superfície corporal; FAC: variação da área fracionada; FE: fração de ejeção; SLG: strain longitudinal global; SLPLVD: strain longitudinal da parede livre do ventrículo direito; VD: ventrículo direito; VE: ventrículo esquerdo.

**Figura 2:**
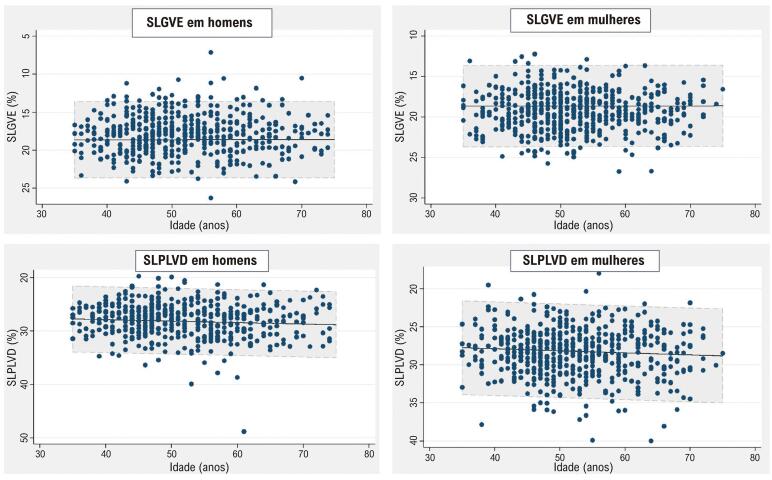
– Valores de SLGVE e SLPLVD entre idades em homens e mulheres na subamostra saudável. SLGVE: strain longitudinal global do ventrículo esquerdo; SLPLVD: strain longitudinal da parede livre do ventrículo direito.

Entre os 527 participantes classificados como saudáveis (50,2 anos; 59% mulheres), a SLGVE média foi de 19,0% (IC 95%: 14,3% a 23,8%), enquanto o valor médio de SLPLVD foi de 28,3% (IC 95%: 22,3% a 34,3%). As mulheres exibiram valores absolutos mais altos de SLGVE e SLPLVD (Tabela 3). No entanto, não houve diferenças nos valores de SLGVE e SLPLVD entre diferentes faixas etárias ou na análise estratificada por sexo (Figura 2). A Tabela 4 apresenta as características clínicas e ecocardiográficas da subamostra saudável estratificada por sexo.

**Tabela 3:** – Valores de referência do strain longitudinal da subamostra saudável

	n	Média ± DP	Limites do normal (IC 95%)	Valor p
**SLGVE,%**				
Geral	527	19,0 ± 2,4	14 a 24	-
Homens	215	18,3 ± 2,3	14 a 23	<0,01
Mulheres	312	19,5 ± 2,3	15 a 24	
**SLPLVD, %**				
Geral	527	28,3 ± 3,1	22 a 34	-
Homens	215	28,1 ± 3,1	22 a 34	0,07
Mulheres	312	28,5 ± 3,0	23 a 34	

DP: desvio padrão; IC: intervalo de confiança; SLGVE: strain longitudinal global do ventrículo esquerdo; SLPLVD: strain longitudinal da parede livre do ventrículo direito.

**Tabela 4:** – Características clínicas e ecocardiográficas basais da subamostra saudável

	Geral (n = 527)	Homens (n = 215)	Mulheres (n = 312)	Valor p
**Idade, anos**	50,2 ± 8,1	49,6 ± 8,4	50,6 ± 7,9	0,13
**Raça autodeclarada, n (%)**				
Branca	291 (55)	116 (53)	175 (56)	0,23
Preta	75 (14)	25 (12)	50 (16)	0,15
Parda	137 (26)	63 (29)	74 (23,7)	0,15
Indígena	8 (2)	4 (2)	4 (1)	0,59
Asiática	13 (2)	7 (3)	6 (2)	0,33
**Altura, cm**	164,9 ± 9,3	172,9 ± 6,8	159,4 ± 6,4	<0,01
**ASC, m^2^**	1,76 ± 0,2	1,91 ± 0,17	1,67 ± 0,15	<0,01
**IMC, kg/m^2^**	25,7 ± 3,99	25,9 ± 3,76	25,5 ± 4,14	0,3
**Parâmetros ecocardiográficos**				
**Diâmetro do AE, cm**	3,4 ± 0,4	3,6 ± 0,4	3,3 ± 0,4	<0,01
**LA volume, mL**	47,5 ± 13	51,3 ± 13,7	45,2 ± 12,1	<0,01
**Volume do AE indexado à ASC, mL/m2**	26,8 ± 6,3	26,7 ± 6,4	26,9 ± 6,2	0,7
**Diâmetro diastólico do VE, cm**	4,4 ± 0,4	4,7 ± 0,4	4,3 ± 0,4	<0,01
**Diâmetro sistólico do VE, cm**	2,8 ± 0,4	3,0 ± 0,4	2,7 ± 0,3	<0,01
**Volume diastólico final do VE, mL**	89,9 ± 18,1	100 ± 17,7	83 ± 14,8	<0,01
**Volume diastólico final do VE indexado à ASC, mL/m2**	51,7 ± 10,2	54,4 ± 10,9	49,9 ± 9,3	<0,01
**Volume sistólico final do VE, mL**	29,6 ± 8,5	33,4 ± 9,9	27,2 ± 6,4	<0,01
**Volume sistólico final do VE indexado à ASC, mL/m2**	17,2 ± 5,7	18,5 ± 6,9	16,3 ± 4,5	<0,01
**Massa do VE indexada à ASC, g/m^2^**	70,8 ± 14,5	77,4 ± 13,7	66,4 ± 13,4	<0,01
**LV massa/altura2,7, g/m2,7**	32,4 ± 7,2	33,6 ± 6,8	31,6 ± 7,3	<0,01
**Espessura relativa da parede**	0,39 ± 0,05	0,39 ± 0,05	0,39 ± 0,06	0,5
**Relação E/e’ mitral**	6,8 ± 1,6	6,5 ± 1,6	7,1 ± 1,6	<0,01
**Média do anel mitral e’, cm/s**	10,8 ± 2,2	10,5 ± 2,1	10,9 ± 2,3	0,06
**Diâmetro basal do VD, cm**	3,5 ± 0,4	3,7 ± 0,3	3,3 ± 0,3	<0,01
**FEVE (método de Simpson), %**	60,2 ± 6,1	59,4 ± 6,1	60,7 ± 6,0	0,01
**SLGVE, %**	19,0 ± 2,4	18,3 ± 2,3	19,5 ± 2,3	<0,01
Branca (n = 291)	18,9 ± 2,4	18,1 ± 2,2	19,5 ± 2,4	<0,01
Preta (n = 75)	19,2 ± 1,8	19 ± 2,5	19,3 ± 1,8	0,53
Parda (n = 137)	19 ± 2,5	18,2 ± 2,3	19,7 ± 2,4	<0,01
Indígena (n = 8)	19 ± 2,2	19 ± 3,2	19 ± 1	0,99
Asiática (n = 13)	19,2 ± 2,1	18,3 ± 2	20,2 ± 1,9	0,1
**FAC do VD, %**	43,8 ± 5,7	42,8 ± 5,7	44,5 ± 5,6	<0,01
**SLPLVD, %**	28,3 ± 3,1	28,1 ± 3,1	28,5 ± 3,0	0,07
Branca (n = 291)	28,5 ± 3,1	27,9 ± 3,1	28,8 ± 3,1	0,01
Preta (n = 75)	28,2 ± 2,9	28,2 ± 3	28,3 ± 2,9	0,95
Parda (n = 137)	28 ± 3	28 ± 3,2	28 ± 2,7	0,9
Indígena (n = 8)	29,3 ± 2,2	29,5 ± 2,5	29,2 ± 2,2	0,85
Asiática (n = 13)	28 ± 2,4	27,8 ± 2,6	28,2 ± 2,4	0,77

Os números representam média ± desvio padrão. AE: átrio esquerdo; ASC: área de superfície corporal; FAC: variação da área fracionada; FE: fração de ejeção; IMC: índice de massa corporal; SLG: strain longitudinal global; SLPLVD: strain longitudinal da parede livre do ventrículo direito; VD: ventrículo direito; VE: ventrículo esquerdo.

**Figura 3:**
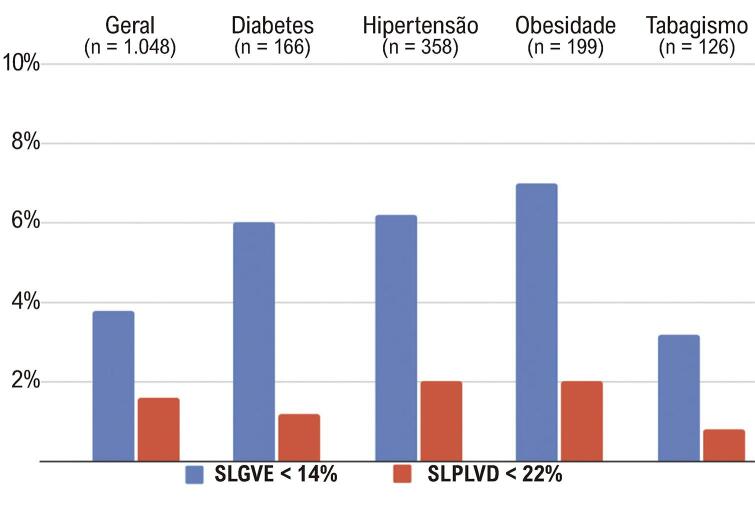
– Distribuição de SLGVE e SLPLVD anormais entre diferentes características clínicas na população do estudo. SLGVE: strain longitudinal global do ventrículo esquerdo; SLPLVD: strain longitudinal da parede livre do ventrículo direito.

SLGVE anormal (< 14%) foi detectado em 3,8% de todos os participantes, enquanto SLPLVD anormal (< 22%) foi encontrado em 1,6%. A Figura Suplementar 1 mostra a distribuição de SLGVE e SLPLVD entre a população do estudo. Taxas mais altas de SLGVE anormal, mas não de SLPLVD, foram observadas em participantes com hipertensão e obesidade (Figura 3; Tabela Suplementar 1).

**Figura 4:**
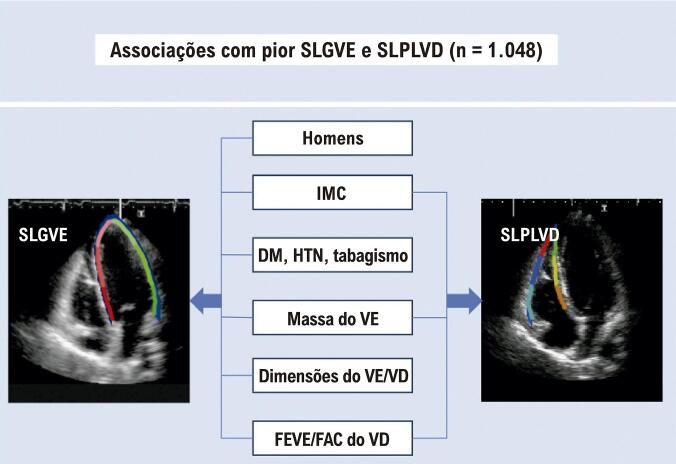
– Características clínicas e ecocardiográficas associadas a pior SLGVE e SLPLVD na população do estudo. DM: diabetes mellitus; FAC: variação da área fracionada; FEVE: fração de ejeção do ventrículo esquerdo; HTN: hipertensão; IMC: índice de massa corporal; SLGVE: strain longitudinal global do ventrículo esquerdo; SLPLVD: strain longitudinal da parede livre do ventrículo direito; VD: ventrículo direito; VE: ventrículo esquerdo.

Na análise de tercil, os indivíduos no grupo de tercil com pior SLGVE eram predominantemente homens e tinham taxas mais altas de hipertensão, diabetes e obesidade, bem como pontuações mais altas de risco cardiovascular. Adicionalmente, esses participantes apresentaram maiores dimensões do VE e VD, maior massa do VE, maior espessura relativa da parede e maior proporção de hipertrofia concêntrica do VE. Ademais, pior SLGVE foi associado a parâmetros sistólicos do VE e do VD mais desfavoráveis, conforme avaliado por FEVE, FAC do VD e SLPLVD (Tabela 5). Em relação aos tercis de SLPLVD, os participantes com pior SLPLVD apresentaram maior índice de massa corporal (IMC), aumento da massa do VE e piores parâmetros sistólicos ventriculares do VE e VD, conforme avaliados por FEVE, SLGVE e FAC do VD (Tabela 6). A Figura 4 ilustra os fatores correlacionados com SLGVE e SLPLVD anormais. Os principais achados do nosso estudo são apresentados na Figura Central.

**Tabela 5:** – Tercis de strain longitudinal global do ventrículo esquerdo da população do estudo

	SLGVE			Valor p
	Melhor ←		Pior →	

	Tercil 1 (n = 350) 26,73% a 19,71%	Tercil 2 (n = 349) 19,71% a 17,64%	Tercil 3 (n = 349) 17,63% a 7,12%	
Parâmetros demográficos				
**Idade, anos**	51,9 ± 8,6	52,1 ± 8,9	51,9 ± 8,6	0,91
**Mulheres, n (%)**	230 (65)	191 (54)	138 (39)	<0,01
**IMC, kg/m2**	25,9 ± 4	26,4 ± 4,2	27,4 ± 4,6	<0,01
**Hipertensão, n (%)**	100 (28,5)	114 (32,6)	144 (41,2)	<0,01
**Diabetes, n (%)**	40 (11,4)	53 (15,1)	73 (20,9)	<0,01
**Tabagismo atual, n (%)**	37 (10,5)	43 (12,3)	46 (13,2)	0,01
**Obesidade, n (%)**	53 (15,1)	59 (16,9)	87 (24,9)	<0,01
**Sobrepeso, n (%)**	142 (40,5)	146 (41,8)	158 (45,2)	0,42
**Doença renal crônica, n (%)**	24 (6,8)	30 (8,5)	38 (10,8)	0,16
**Doença pulmonar obstrutiva crônica, n (%)**	3 (0,8)	7 (2)	5 (1,4)	0,44
**Acidente vascular cerebral, n (%)**	4 (1,1)	3 (0,8)	3 (0,8)	0,9
**Insuficiência cardíaca, n (%)**	3 (0,8)	2 (0,5)	9 (2,5)	0,04
**Infarto do miocárdio prévio, n (%)**	7 (2)	7 (2)	6 (1,7)	0,9
**Pontuação de risco de DCVA, %**	1,95 [0,82 a 5,46]	2,7[1,1 a 6,85]	4 [1,4 a 9,3]	<0,01
**Parâmetros ecocardiográficos**				
**Volume do AE, mL**	47,8 ± 12,04	47,9 ± 13,4	48,3 ± 15,4	0,92
**Volume do AE indexado à ASC, mL/m2**	27,3 ± 6,3	26,7 ± 6,5	25,9 ± 7,3	0,02
**Diâmetro diastólico do VE, cm**	4,4 ± 0,4	4,4 ± 0,4	4,5 ± 0,5	<0,01
**Massa do VE, g**	126,8 ± 31,1	132,1 ± 35,5	147 ± 43,7	<0,01
**Massa do VE indexada à ASC, g/m2**	72,2 ± 14,7	73,5 ± 16,1	79,1 ± 19,3	<0,01
**Massa do VE/altura2,7, g/m2,7**	33,5 ± 7,4	34 ± 8,3	36,6 ± 9,8	<0,01
**Padrões geométricos do VE, n (%)**				
Remodelamento concêntrico	98 (28)	95 (27,2)	113 (32,3)	0,27
Hipertrofia concêntrica	9 (2,6)	15 (4,3)	25 (7,2)	0,01
Hipertrofia excêntrica	20 (5,7)	11 (3,1)	17 (4,9)	0,25
**Espessura relativa da parede**	0,39 ± 0,062	0,4 ± 0,067	0,42 ± 0,074	<0,01
**Relação E/e’ mitral**	7,4 ± 1,8	7,1 ± 1,9	7,3 ± 1,9	0,3
**Média do anel mitral e’, cm/s**	10,6 ± 2,3	10,3 ± 2,5	9,3 ± 2,3	0,32
**FEVE (método de Simpson), %**	63,9 ± 5,1	59,8 ± 4,5	54,7 ± 5,4	<0,01
**Diâmetro basal do VD, cm**	3,5 ± 0,3	3,5 ± 0,4	3,5 ± 0,4	0,01
**FAC do VD, %**	44,1 ± 5,5	43,8 ± 5,6	43,4 ± 5,7	0,21
**SLPLVD, %**	28,7 ± 3	28,1 ± 3,3	27,8 ± 3,2	<0,01

Os números representam média ± desvio padrão para variáveis contínuas com distribuição normal, mediana [intervalo interquartil] para variáveis contínuas com distribuição anormal e n (%) para variáveis categóricas. As variáveis contínuas foram comparadas usando um teste t bilateral com variância desigual para dados com distribuição normal, o teste de postos sinalizados de Wilcoxon para dados com distribuição anormal e teste qui-quadrado para variáveis categóricas. AE: átrio esquerdo; ASC: área de superfície corporal; DCVA: doença cardiovascular aterosclerótica; FAC: variação da área fracionada; FE: fração de ejeção; IMC: índice de massa corporal; SLG: strain longitudinal global; SLPLVD: strain longitudinal da parede livre do ventrículo direito; VD: ventrículo direito; VE: ventrículo esquerdo.

**Tabela 6:** – Tercis de strain longitudinal da parede livre do ventrículo direito da população do estudo

	SLPLVD			
	Melhor		Pior	Valor p
	←		→	
	Tercil 1 (n = 350) 48,8% a 29,37%	Tercil 2 (n = 349) 29,36% a 26,77%	Tercil 3 (n = 349) 23,76% a 17,96%	
Parâmetros demográficos				
**Idade, anos**	53 ± 8,8	51,3 ± 8,2	51,7 ± 9	<0,01
**Mulheres, n (%)**	197 (56)	192 (55)	170 (48)	0,1
**IMC, kg/m2**	26,1 ± 4	26,5 ± 4,7	27,1 ± 4,7	<0,01
**Hipertensão, n (%)**	110 (31,4)	125 (35,8)	123 (35,2)	0,44
**Diabetes, n (%)**	54 (15,4)	56 (16)	56 (16)	0,96
**Tabagismo atual, n (%)**	46 (13,1)	38 (10,9)	42 (12)	0,62
**Obesidade, n (%)**	56 (16)	64 (18,3)	79 (22,6)	0,07
**Sobrepeso, n (%)**	139 (39,7)	160 (45,8)	147 (42,1)	0,25
**Doença renal crônica, n (%)**	27 (7,7)	28 (8)	37 (10,5)	0,33
**Doença pulmonar obstrutiva crônica, n (%)**	4 (1,1)	6 (1,7)	5 (1,4)	0,81
**Acidente vascular cerebral, n (%)**	2 (0,6)	4 (1,1)	4 (1,1)	0,66
**Insuficiência cardíaca, n (%)**	4 (1,1)	7 (2)	3 (0,8)	0,38
**Infarto do miocárdio prévio, n (%)**	4 (1,1)	8 (2,3)	8 (2,3)	0,43
**Pontuação de risco de DCVA, %**	3,3 [1,1 a 7,6]	2,3 [1,1 a 6,1]	2,9 [1,1 a 6,9]	0,37
**Parâmetros ecocardiográficos**				
**Volume do AE, mL**	48,6 ± 14,3	47,5 ± 12,4	47,9 ± 14,1	0,58
**Volume do AE indexado à ASC, mL/m2**	27,2 ± 7,2	26,3 ± 6	26,4 ± 6,9	<0,01
**Diâmetro diastólico do VE, cm**	4,5 ± 0,4	4,5 ± 0,5	4,4 ± 0,5	0,64
**Massa do VE, g**	133,1 ± 37,7	137,1 ± 38,8	135,2 ± 37,3	<0,01
**Massa do VE indexada à ASC, g/m2**	74,3 ± 17	76 ± 17,5	74,3 ± 16,3	0,34
**Massa do VE/altura2,7, g/m2,7**	34,2 ± 8,6	35,2 ± 8,6	34,7 ± 8,7	0,97
**Padrões geométricos do VE, n (%)**				
Remodelamento concêntrico	91 (26)	103 (29,5)	112 (32)	0,2
Hipertrofia concêntrica	17 (4,8)	18 (5,1)	14 (4)	0,75
Hipertrofia excêntrica	16 (4,6)	19 (5,4)	13 (3,7)	0,55
**Espessura relativa da parede**	0,40 ± 0,067	0,41 ± 0,071	0,41 ± 0,066	0,23
**Relação E/e’ mitral**	7,4 ± 1,9	7,2 ± 1,8	7,1 ± 1,9	0,21
**Média do anel mitral e’, cm/s**	10,1 ± 2,4	10,2 ± 2,4	9,9 ± 2,4	0,86
**FEVE (método de Simpson), %**	60,2 ± 6,2	59,5 ± 6,5	58,7 ± 6,1	<0,01
**SLGVE, %**	19 ± 2,5	18,6 ± 2,6	18,3 ± 2,5	<0,01
**Diâmetro basal do VD, mm**	35,2 ± 3,7	34,9 ± 4,3	34,8 ± 3,7	0,3
**FAC do VD, %**	45,6 ± 5,4	44 ± 5,3	41,8 ± 5,6	<0,01

Os números representam média ± desvio padrão para variáveis contínuas com distribuição normal, mediana [intervalo interquartil] para variáveis contínuas com distribuição anormal e n (%) para variáveis categóricas. As variáveis contínuas foram comparadas usando um teste t bilateral com variância desigual para dados com distribuição normal, o teste de postos sinalizados de Wilcoxon para dados com distribuição anormal e teste qui-quadrado para variáveis categóricas. AE: átrio esquerdo; ASC: área de superfície corporal; DCVA: doença cardiovascular aterosclerótica; FAC: variação da área fracionada; FE: fração de ejeção; IMC: índice de massa corporal; SLG: strain longitudinal global; SLPLVD: strain longitudinal da parede livre do ventrículo direito; VD: ventrículo direito; VE: ventrículo esquerdo.

A análise de regressão revelou uma associação significativa entre FEVE e SLGVE (r = −0,66, p < 0,01), bem como entre SLPLVD e FAC do VD (r = −0,31, p < 0,01) na população total. Associações semelhantes foram observadas na população saudável (r = −0,65, p < 0,01; r = −0,32, p < 0,01, respectivamente), conforme ilustrado na Figura Suplementar 2.

## Discussão

Até onde sabemos, esta é a maior coorte brasileira usada para estimar valores de referência para SLGVE e SLPLVD em uma população adulta multiétnica. Nossos achados indicam diferenças significativas em SLGVE e SLPLVD de acordo com o sexo, mas não com a idade. Verificamos que os indivíduos com valores absolutos reduzidos de SLGVE eram predominantemente homens, com maiores taxas de hipertensão, diabetes e obesidade; eles exibiam maiores dimensões ventriculares, pior hipertrofia concêntrica do VE e piores parâmetros sistólicos de FEVE e VD. Ademais, os participantes com valores absolutos reduzidos de SLPLVD tinham IMC e massa de VE mais altos, juntamente com pior FEVE e FAC do VD. Esses achados sugerem uma interação complexa entre fatores demográficos, clínicos e ecocardiográficos que influenciam o SLGVE e o SLPLVD.

O valor médio de referência de SLGVE relatado em nosso estudo foi semelhante ao de outros estudos. Uma metanálise publicada, que incluiu mais de 2.500 participantes de 24 estudos, relatou um valor normal de SLGVE de 19,7% (IC 95%: 18,9% a 20,4%),^15^ e um estudo transversal menor recente, incluindo participantes brasileiros saudáveis (n = 77), apresentou 19% ± 2% como valor de referência.^16^ Nosso valor médio absoluto de referência para o SLPLVD foi semelhante a alguns estudos,8 mas maior em comparação a outros. Em um estudo de coorte prospectivo dinamarquês de pacientes sem doenças cardiovasculares ou fatores de risco, o valor médio de SLPLVD foi de 26,7% ± 5,2%.17 Nyberg et al.^18^ publicaram valores de referência para SLG em uma coorte norueguesa, mostrando um valor médio de SLPLVD de 25,9% (IC 95%: 17,4% a 34,5%). Outro estudo em indivíduos saudáveis realizado na Índia demonstrou um valor médio de SLPLVD de 23,6% ± 3,8%.^19^ Explicações potenciais para a disparidade nesses resultados podem ser atribuídas a diferenças relacionadas à etnia e à utilização de software distinto para análise de strain do VD. Nossos dados sugerem que os valores de referência para essas medidas ecocardiográficas podem variar significativamente entre as populações, e a aplicação de valores globais pode ser imprecisa.

Há evidências crescentes sobre o impacto do sexo e da idade no SLGVE,20 mesmo quando esse parâmetro foi obtido usando outros métodos, como ressonância magnética cardíaca.21 Em nosso estudo, o valor absoluto de SLGVE foi 1,3% maior em mulheres em comparação aos homens, o que é consistente com valores relatados anteriormente em uma população geral sem doença cardiovascular ou fatores de risco tradicionais.16 Ademais, reafirmamos o efeito do sexo na deformação miocárdica do VE expressa por uma menor prevalência de mulheres no tercil mais baixo do SLGVE. Em relação ao SLPLVD, observamos uma tendência a valores absolutos mais altos entre as mulheres em nossa amostra. As diferenças relacionadas ao sexo na função sistólica podem ser explicadas por diferenças estruturais observadas em mulheres, caracterizadas por cavidades menores. No entanto, fatores neuro-hormonais e outros fatores biológicos também podem influenciar esse processo, particularmente entre mulheres com menos de 60 anos de idade.22 Os efeitos da idade na deformação miocárdica continuam sendo um tópico de controvérsia. No estudo de coorte conduzido por Sengupta et al.,19 envolvendo voluntários saudáveis, não foi observada disparidade relacionada à idade em SLGVE. Além disso, de acordo com os achados do estudo de coorte de Espersen et al.,17 a idade não teve uma associação independente com SLPLVD na análise de regressão linear multivariável. Dentro de nossa coorte, a idade não demonstrou uma associação significativa com o SLG. No entanto, vale notar que a faixa etária relativamente estreita em nossa amostra pode ter limitado nossa capacidade de detectar diferenças em SLG em diversas faixas etárias.

Nosso estudo encontrou uma proporção significativamente maior de valores anormais de SLGVE em participantes com fatores de risco para doenças cardiovasculares em comparação com aqueles sem tais fatores de risco. Em contraste, não houve diferença estatisticamente significativa na proporção de valores anormais de SLPLVD entre participantes com e sem fatores de risco para doenças cardiovasculares. Ademais, pior SLGVE foi associado a fatores de risco cardiovascular, como hipertensão, diabetes e obesidade, semelhante aos achados relatados em outros estudos.^23^ Na hipertensão, foi descrita redução nos valores do SLGVE mesmo nos estágios iniciais do espectro da doença, com diferenças detectadas entre pré-hipertensão e hipertensão estágio I (SLGVE 17,5% ± 2,5% versus 18,2% ± 2,4%, p = 0,03), conforme demonstrado em um estudo brasileiro.^24^ Indivíduos com diabetes também apresentaram piores valores de SLGVE,^25^ e um estudo recente mostrou que tratamento com hipoglicemiantes (inibidores do transportador de sódio-glicose 2) melhorou esse parâmetro, mesmo após um curto período de acompanhamento.26 Adicionalmente, a obesidade foi associada à redução subclínica da deformação miocárdica medida pelo strain do VE e VD, mesmo em indivíduos sem qualquer doença cardiovascular, e o grau de elevação do IMC foi associado a um risco incremental de disfunção miocárdica subclínica.^27^ Outros fatores de risco cardiovascular, além do IMC, não foram associados a pior SLPLVD. O comprometimento funcional do VD é provavelmente multifatorial, atribuível tanto às condições que levam à sobrecarga de pressão do VD devido à pressão pulmonar elevada quanto à sobrecarga de volume resultante da disfunção do VE, culminando em insuficiência biventricular.^28^ Essa proposição sugere que o strain do VD pode ter maior relevância clínica em contextos específicos da doença envolvendo predominantemente as câmaras do lado direito, em vez de demonstrar utilidade na população geral com baixo risco cardiovascular.

Considerando os achados ecocardiográficos, nosso estudo revelou que pior SLGVE e SLPLVD estavam associados à hipertrofia do VE, conforme indicado pelo aumento da massa do VE, reforçando a hipótese de que a hipertrofia do VE pode induzir uma alteração progressiva nas fibras miocárdicas subendocárdicas e subepicárdicas, inicialmente caracterizada pela atenuação do strain longitudinal.^29 ^Ademais, maiores dimensões ventriculares estavam associadas a pior SLGVE, sugerindo uma maior suscetibilidade da camada endocárdica a lesões hemodinâmicas. Yoshida et al. propõem vários mecanismos para explicar a associação independente entre o SLG e a disfunção do AE e do VD. Um mecanismo proposto é a microcirculação coronária prejudicada, que pode afetar negativamente a função ventricular e atrial. Adicionalmente, interações anatômicas podem explicar essas relações. A função do VE modula a função do reservatório do AE por meio do movimento descendente sistólico da base do VE. Em relação à interdependência ventricular, o VE e o VD compartilham miofibras que circundam ambos os ventrículos.^30^ A associação entre o strain do VE e do VD e outros parâmetros tradicionais da função do VE e do VD (FEVE e FAC do VD) destaca a interdependência ventricular e a forte conexão entre as câmaras do VE e do VD.^17^

### Limitações

Algumas limitações da presente análise devem ser observadas. As imagens ecocardiográficas foram adquiridas entre 2008 e 2010, seguindo as diretrizes em vigor na época. A análise de strain não foi inicialmente prevista ou incorporada ao protocolo original. Como resultado, nossa análise de SLGVE foi baseada na avaliação offline de janelas apicais de 4 e 2 câmaras. Consequentemente, usamos o modelo de 12 segmentos para análise do SLGVE, que era comumente aplicado em outras populações de grande escala.^31,32^ Ademais, nossos achados de strain devem ser interpretados considerando que as imagens foram analisadas usando software independente de fornecedor, e os valores normais relatados podem não ser diretamente transferíveis para medidas realizadas usando software específico do fornecedor. Embora a ecocardiografia tridimensional com speckle-tracking tenha o potencial para uma avaliação mais precisa da função miocárdica,^33^ o protocolo do ELSA-Brasil exigiu apenas imagens bidimensionais. Outra limitação do presente estudo é o baixo coeficiente de correlação intraclasse interobservador para as medidas de SLGVE e SLPLVD, semelhante a estudos anteriores que podem refletir a curva de aprendizado da análise de strain, especialmente para strain do VD. A variabilidade interobservador, particularmente para SLGVE, tem sido documentada na literatura e pode resultar de diferenças na experiência do observador, qualidade da imagem e uso de software. Além disso, o estudo está sujeito a potencial viés de seleção devido ao uso de uma coorte de servidores públicos de universidades e instituições de pesquisa, que pode não ser totalmente representativa da população brasileira em geral. Finalmente, nossa estreita faixa etária pode limitar a generalização desses achados para outras faixas etárias.

## Conclusão

Nosso estudo dentro da grande população brasileira, de meia-idade e multiétnica da coorte ELSA-Brasil fornece conhecimentos valiosos sobre os valores de referência de SLG para o VE e o VD. Ademais, fomos capazes de demonstrar que o SLGVE e o SLPLVD reduzidos estavam associados a comorbidades cardiovasculares, estrutura e função cardíaca, usando parâmetros ecocardiográficos frequentemente empregados, especialmente SLGVE.
