# Supplementary material for: Strain Ventricular em uma População Adulta Brasileira do Estudo ELSA-Brasil: Valores de Referência e seus Determinantes
Source: Arq Bras Cardiol. 2025 May 30;122(6):e20240634. [Article in Portuguese] doi: 10.36660/abc.20240634 (PMC12217928; doi:10.36660/abc.20240634)
Supplement: Translation

# Translation: Left and Right Ventricular Strain in an Adult Brazilian Population from ELSA-Brasil Study: Reference Values and their Determinants

**DOI:** 10.36660/abc.20240634i

**Authors:** Eduardo Gatti Pianca, Murilo Foppa, Giulia Bevilacqua Schmitz, Wilson Cañon-Montañez, Bruce Bartholow Duncan, Angela Barreto Santiago Santos

**Affiliations:** 1 Brazil Universidade Federal do Rio Grande do Sul – Programa de Pós-Graduação em Cardiologia e Ciências Cardiovasculares, Faculdade de Medicina, Porto Alegre, RS – Brazil; 2 Brazil Hospital de Clinicas de Porto Alegre, Porto Alegre, RS – Brazil; 3 Colombia Universidad de Antioquia – Faculty of Nursing, Medellin – Colombia

**Keywords:** Global Longitudinal Strain, Heart Ventricles, Cohort Studies, Echocardiography, Ventricular Function

## Abstract

**Background:**

Impaired left ventricular (LV) and right ventricular functions are important predictors of cardiovascular risk. Global longitudinal strain (GLS) provides superior sensitivity for assessing systolic function compared to traditional parameters, enhancing diagnostic accuracy in various cardiac conditions. However, GLS reference values in diverse populations are lacking.

**Objectives:**

To establish reference values for LVGLS and right ventricular free wall longitudinal strain (RVFWLS) in a Brazilian multiethnic population without cardiovascular risk factors or disease. We also explore how clinical and echocardiographic factors influence GLS distribution, addressing a gap in global guidelines that often rely on data from homogeneous or geographically distant populations.

**Methods:**

We included 1,048 participants from the ELSA-Brasil cohort who underwent echocardiography with GLS analysis. A healthy subsample (n = 527) was defined by excluding individuals with cardiovascular or renal disease, hypertension, or diabetes to establish GLS reference ranges. The prevalence of abnormal GLS was assessed, and factors associated with reduced LVGLS and RVFWLS were identified. Statistical significance was defined as p < 0.05.

**Results:**

In the healthy subsample (mean age 50.2 years, 59% female), mean LVGLS was 19.0% (95% confidence interval: 14.3 to 23.8) and RVFWLS was 28.3% (95% confidence interval: 22.3 to 34.3). Females exhibited higher LVGLS and RVFWLS values than males, with no significant age-related differences. Abnormal LVGLS and RVFWLS were observed in 3.8% and 1.6% of participants, respectively. Lower LVGLS was associated with obesity, hypertension, and diabetes; reduced RVFWLS correlated with higher body mass index and LV mass.

**Conclusions:**

We propose reference values for LVGLS and RVFWLS in a large Brazilian cohort, highlighting associations with cardiovascular comorbidities and ventricular structure.

## Introduction

Impaired left ventricular (LV) systolic function is a well-known predictor of cardiovascular morbidity and mortality,1 and it is usually reported and categorized by the left ventricular ejection fraction (LVEF). However, LVEF has recognized reproducibility and standardization limitations, decreasing the detection of subtle abnormalities in LV function.^2^ LV contractility assessment through myocardial strain using speckle tracking has overcome most of these restrictions. Furthermore, this technique is resilient to variations in acquisition planes and direction of muscle movement, thus allowing the evaluation in regular bidimensional echocardiographic images.^3^ Particularly, global longitudinal strain (GLS) analysis has been shown a robust measure to detect early systolic dysfunction and a prognostic parameter beyond LVEF.^4,5^

Right ventricular (RV) failure is also emerging as a prognostic parameter in scenarios such as heart failure, myocardial infarction, and pulmonary hypertension.^6^ Nonetheless, the assessment of RV function through conventional echocardiography such as tricuspid lateral annular peak systolic velocity by pulsed tissue Doppler imaging (s’) and tricuspid annular plane systolic excursion (TAPSE) is limited due to the complex shape of this chamber and the evaluation restricted to the basal segments rather than the entire RV. To address this challenge, emerging parameters such as RV strain have proven to be reliable and accurate with the advantage of being less affected by angle dependency than traditional function parameters.^6^ Moreover, RV longitudinal strain has enhanced diagnostic accuracy across various heart conditions, including pulmonary hypertension, pulmonary embolism, and arrhythmogenic RV dysplasia.^6^ Additionally, it aids in further stratifying the prognosis among patients with heart failure, acute coronary syndromes, and heart transplantation.^6^

Despite its strengths, LVGLS and right ventricular free wall longitudinal strain (RVFWLS) measurements have several limitations that should be considered when interpreting results in both clinical practice and research. These limitations primarily stem from issues related to image quality (such as patient body habitus, poor acoustic windows, or motion artifacts, all of which can significantly impair strain analysis), heart rate variability (where very high or low heart rates can affect the timing of myocardial contraction and strain measurement, potentially distorting the results), and software variability (as different commercial software platforms may employ slightly different algorithms for strain calculation, leading to variability in GLS measurements). Additionally, RVFWLS is inherently more challenging to assess due to the complex anatomy and function of the RV. Understanding these limitations is essential for accurate interpretation of strain measurements and for preventing over-interpretation of results, particularly in patients with borderline or subtle abnormalities.^7^

The American Society of Echocardiography and the European Association of Echocardiography have established standard reference values for echocardiography measurements.^8^ However, these data may not accurately represent diverse world populations, as already shown in previous studies including LVEF.^9^ Although global populations may serve as a starting point, the genetic, social, and environmental variations between countries and continents suggest that universal reference values might be imprecise for clinical diagnosis. The need for local reference values becomes even more relevant in countries with a multiethnic population, like Brazil, where genetic and behavioral characteristics can vary considerably between different ethnic groups. To ensure a more accurate assessment of ventricular function in patients from this population, it is crucial to define reference values specific to Brazil, based on the characteristics of the local population. The ELSA-Brasil Study presents a unique opportunity to establish reference limits for GLS parameters from a large Brazilian adult sample, allowing better interpretation of echocardiography results in our population while the implementation of these parameters happens in daily clinical practice. Based on a middle-aged Brazilian multiethnic population, we aimed to: 1) describe reference values for LVGLS and RVFWLS in a subsample free of cardiovascular disease and risk factors; 2) describe the prevalence of abnormal GLS in this occupational cohort, using the healthy subsample cutoff; 3) evaluate the influence of clinical and echocardiographic factors on GLS distribution within this occupational cohort.

## Methods

### Study population

The Brazilian Longitudinal Study of Adult Health (ELSA-Brasil) is a prospective epidemiologic study designed to investigate cardiovascular disease and diabetes in 15,105 participants who are civil servants from universities or research institutions in 6 Brazilian cities (São Paulo, Rio de Janeiro, Belo Horizonte, Vitória, Salvador, and Porto Alegre). All active or retired employees aged 35 to 74 years were eligible for the study. The details of the study, including design, eligibility criteria, sources and methods of recruitment, and measurements obtained have been described elsewhere.10,11 The present investigation was a cross-sectional study of the ELSA-Brasil during the first visit (August 2008 to December 2010), from a predefined random subsample comprising 10% of the total sample. From this sample, we included participants who underwent an echocardiographic exam with suitable LV and RV analysis and were in sinus rhythm during the examination. The analysis for the definition of LVGLS and RVFWLS reference values was restricted to a subsample of “healthy” participants composed of those free of hypertension; diabetes; renal disease, defined as a glomerular filtration rate < 60 mL/min/1.73 m^2^ or urine albumin/creatinine ratio ≥ 30 mg/g; and preexisting cardiovascular conditions, which were defined as history of stroke, heart failure, or myocardial infarction (Figure 1).

**Figure 1:** – Flowchart of the study. DM: diabetes mellitus; HTN: hypertension; LV: left ventricle; RV: right ventricle.

The ELSA-Brasil Study was approved by the research and ethics committees of the institutions involved, and all participants provided written informed consent.

### Echocardiography

All transthoracic echocardiograms were obtained at the first study visit at 6 different centers. Images were acquired on identically configured echocardiography machines (Aplio XG, Toshiba) with a 2.5 MHz sector transducer, and the frame rate for the acquisition ranged from 40 to 60 frames per second, according to standard protocols. These protocols included the parasternal long and short axis views, as well as the apical 4-chamber and 2-chamber views. The exams were recorded in digital format and transferred to the ELSA-Brasil Echo reading center in Porto Alegre, Rio Grande do Sul. Standard echocardiographic and Doppler parameters were analyzed using an offline workstation (ComPACS 10.5 workstation; Medimatic SrL, Italy). All measurements were made in triplicate following the recommendations of the American Society of Echocardiography,8 including LV diameters, LV wall thickness, LV mass, LVEF, left atrium (LA) volume, early and late diastolic mitral inflow velocities, mitral annular velocities, RV diameter, RV diastolic and systolic areas, and RV fractional area change (FAC).

To evaluate the parameters of early systolic dysfunction, we followed current guidelines and recommendations.12 The LV and RV myocardial deformation function was measured using a previously validated and commercially available software dedicated to LV and RV analysis (2D Cardiac Performance Analysis©, TomTec-ArenaTM 1.2 Imaging Systems, Unterschleißheim, Germany). For LV strain evaluation, the endocardial borders were traced at the end-diastolic frame of 2-dimensional images acquired from the apical 2- and 4-chamber views. End-diastole was defined by the QRS complex, or as the frame after the mitral valve closure. End-systole was defined by the visualization of the frame before the mitral valve opening in the parasternal long axis view. Speckles were tracked frame by frame within the LV myocardium throughout 1 cardiac cycle; basal, mid, and apical regions of interest were then created. Each image was carefully inspected and the segments that failed to track were manually adjusted. If more than 1 segment could not be tracked, or if there was a lack of a full cardiac cycle or significant LV foreshortening, the measurements were considered unreliable, and the patient was excluded from the analysis. LVGLS was calculated as the average longitudinal strain across the apical 2- and 4-chamber views. For the evaluation of RV longitudinal strain, the end-diastole was manually defined by the QRS complex, whereas end-systole was defined as a tricuspid valve opening from the 4-chamber view. The RV free wall and interventricular septum were both divided into 3 segments (apical, mid, and basal). RVFWLS is the average value from 3 RV free wall segments. All GLS values are presented as absolute values and were obtained during the peak contraction phase of the cardiac cycle (peak systolic strain).

Intra- and inter-observer reproducibility for LVGLS and RVFWLS measurements were previously assessed in 50 randomly selected cases from the ELSA-Brasil cohort. For LVGLS, the intra- and inter-observer coefficients of variation were 5.4% and 7.4%, with intraclass correlation coefficients (ICC) of 0.83 (95% confidence interval [CI]: 0.73 to 0.90) and 0.76 (95% CI: 0.61 to 0.86), respectively.13 For RVFWLS, the coefficients of variation were 5.1% and 8.3%, with ICC values of 0.78 (95% CI: 0.67 to 0.89) and 0.54 (95% CI: 0.34 to 0.74), respectively.^14^

### Statistical analyses

Continuous normally distributed data were presented as mean and standard deviation. Continuous abnormally distributed data were displayed as median and interquartile range, and categorical data were shown as total and proportion. Normality was assessed using the Shapiro-Wilk test. Continuous variables were compared using a 2-sided t test with unequal variance for normally distributed data, the Wilcoxon signed rank test for abnormally distributed data, and chi-squared tests for categorical variables. We presented the mean values and corresponding 95% CI for LVGLS and RVFWLS, defining absolute values below this cutoff in the healthy sample as abnormal. We categorized the sample into tertiles according to the severity of LV and RV systolic function measured by GLS, and applied trend tests (2-sided t test with unequal variance, Wilcoxon signed rank test, and chi-squared tests) to illustrate the association of GLS with demographic characteristics and echocardiographic measurements of cardiac structure and function. Linear regression analysis was performed to assess the association between LVEF and LVGLS, as well as between RVFWLS and RVFAC.

All analyses were performed with the STATA software package (version 13, Stata Corp, College Station, Texas, United States). All tests were 2-sided, and p values < 0.05 were considered statistically significant.

## Results

Our total population (n = 1,048) had a mean age of 52 years and included 559 (53%) females. The largest recruitment center was São Paulo (26.7%), followed by Belo Horizonte (22.6%), Porto Alegre (16.6%), Salvador (13.7%), Rio de Janeiro (13.1%), and Vitória (7.1%). Table 1 presents the clinical characteristics of this study population stratified by sex. Age did not differ between sexes, whereas male subjects had higher body surface area, more prevalent hypertension, diabetes, and current smoking status, as well as worse cardiovascular risk scores. Regarding echocardiographic parameters, females had smaller cavities (LA, LV, and RV diameters), lower LV mass and relative wall thickness, and a higher E/e’ ratio compared to males. Systolic function and myocardial deformation parameters for both LV and RV were higher in females than in males, including higher LVEF, LVGLS, RVFAC, and RVFWLS (Table 2).

**Table 1:** – Baseline clinical characteristics in the study population

	Overall (n = 1,048)	Male (n = 489)	Female (n = 559)	p value
**Age, years**	52 ± 8.7	51.9 ± 9	52.1 ± 8.4	0.77
**Self-identified skin color, n (%)**				
White	523 (49.9)	248 (49.2)	277 (50.4)	0.7
Black	184 (17.5)	79 (16.1)	105 (18.7)	0.26
Brown (pardo)	293 (27.9)	145 (29.6)	148 (26.4)	0.25
Indigenous	13 (1.2)	8 (1.6)	5 (0.9)	0.27
Asian	28 (2.7)	13 (2.6)	15 (2.7)	0.98
**Height, cm**	165.3 ± 9.2	172.1 ± 7.2	159.4 ± 6.2	<0.01
**BSA, m^2^**	1.8 ± 0.2	1.92 ± 0.17	1.59 ± 0.15	<0.01
**BMI, kg/m^2^**	26.6 ± 4.3	26.7 ± 4.1	26.5 ± 4.5	0.43
**Hypertension, n (%)**	358 (34.1)	191 (39)	167 (29.8)	<0.01
**Diabetes, n (%)**	166 (15.8)	100 (20.4)	66 (11.8)	<0.01
**Current smoker, n (%)**	126 (12)	63 (12.8)	63 (11.2)	<0.01
**Obesity, n (%)**	199 (18.9)	86 (17.5)	113 (20.2)	0.27
**Overweight, n (%)**	446 (42)	231 (47.2)	215 (38.4)	<0.01
**Heart failure, n (%)**	14 (1.3)	6 (1.2)	8 (1.4)	0.78
**Previous myocardial infarction, n (%)**	20 (3.6)	12 (1.1)	8 (1.6)	0.22
**Chronic kidney disease, n (%)**	92 (8.7)	56 (11.4)	36 (6.4)	0.13
**Chronic obstructive pulmonary disease, n (%)**	15 (1.4)	4 (0.8)	11 (1.9)	0.11
**Stroke, n (%)**	10 (0.9)	3 (0.6)	7 (1.2)	0.28
**ASCVD risk score (%)**	2.7 [1.1 a 6.8]	5.3 [2.4 a 11]	1.4 [0.7 a 3.7]	<0.01

Numbers represent mean ± standard deviation for continuous variables with normal distribution, median [interquartile range] for continuous variables with abnormal distribution, and n (%) for categorical variables. ASCVD: atherosclerotic cardiovascular disease; BMI: body mass index; BSA: body surface area.

**Table 2:** – Echocardiographic parameters in the study population

	Overall (n = 1,048)	Male (n = 489)	Female (n = 559)	p value
**LA diameter, cm**	3.5 ± 0.4	3.7 ± 0.4	3.4 ± 0.4	<0.01
**LA volume, mL**	48 ± 13.6	51.1 ± 14.6	45.5 ± 12.2	<0.01
**BSA-indexed LA volume index, mL/m2**	26.7 ± 6.7	26.5 ± 6.9	26.8 ± 6.5	0.61
**LV diastolic diameter, cm**	4.5 ± 0.4	4.7 ± 0.4	4.3 ± 0.4	<0.01
**LV systolic diameter, cm**	2.8 ± 0.4	3 ± 0.4	2.7 ± 0.3	<0.01
**LV end-diastolic volume, mL**	91 ± 18.5	99.7 ± 18.2	83.8 ± 15.5	<0.01
**BSA-indexed LV end-diastolic volume, mL/m2**	51.6 ± 10.7	53.9 ± 11.3	49.7 ± 9.7	<0.01
**LV end-systolic volume, mL**	30.8 ± 10.9	35.2 ± 12.3	27.3 ± 8.2	<0.01
**BSA-indexed LV end-systolic volume, mL/m2**	17.1 ± 5.6	18.4 ± 6.3	16.1 ± 4.7	<0.01
**BSA-indexed LV mass, g/m2**	74.9 ± 17	82.7 ± 17.3	69.2 ± 14.5	<0.01
**LV mass/height2.7, g/m2.7**	34.7 ± 8.6	36.2 ± 8.9	33.4 ± 8.1	<0.01
**LV geometric patterns, n (%)**				
Concentric remodeling	306 (29.1)	141 (28.8)	165 (29.5)	0.8
Concentric hypertrophy	49 (4.7)	20 (4.1)	29 (5.2)	0.4
Eccentric hypertrophy	48 (4.6)	18 (3.7)	30 (5.4)	0.19
**Relative wall thickness**	0.4 ± 0.068	0.41 ± 0.069	0.4 ± 0.068	0.03
**Mitral E to e’ ratio**	7.3 ± 1.9	7 ± 1.9	7.5 ± 1.9	<0.01
**Mean mitral annulus e’, cm/s**	10.1 ± 2.4	9.8 ± 2.3	10.3 ± 2.5	<0.01
**RV basal diameter, cm**	3.5 ± 0.4	3.7 ± 0.3	3.3 ± 0.3	<0.01
**LVEF (Simpson method), %**	59.5 ± 6.3	55.6 ± 6.3	60.2 ± 6.2	<0.01
**LVGLS, %**	18.6 ± 2.5	17.9 ± 2.5	19.2 ± 2.4	<0.01
**White (n = 523)**	18.6 ± 2.5	17.9 ± 2.4	19.3 ± 2.4	<0.01
**Black (n = 184)**	18.4 ± 2.6	17.7 ± 2.7	19 ± 2.4	<0.01
**Brown (pardo) (n = 293)**	18.7 ± 2.6	18 ± 2.5	19.4 ± 2.4	<0.01
**Indigenous (n = 13)**	18.7 ± 2.3	18.2 ± 2.8	19.5 ± 1.4	0.3
**Asian (n = 28)**	18.3 ± 2.4	17.7 ± 2.7	18.9 ± 2.2	0.19
**RVFAC, %**	43.8 ± 5.6	43.1 ± 5.7	44.4 ± 5.5	<0.01
**RVFWLS, %**	28.2 ± 3.1	28 ± 3.2	28.4 ± 3.1	0.03
White (n = 523)	28.3 ± 3.1	27.9 ± 3.1	28.6 ± 3.1	0.02
Black (n = 184)	27.9 ± 3.4	28.1 ± 3.7	27.8 ± 3.2	0.6
Brown (pardo) (n = 293)	28 ± 2.9	27.8 ± 2.9	28.2 ± 2.9	0.3
Indigenous (n = 13)	29.5 ± 2.8	29.2 ± 3.1	29.9 ± 2.6	0.6
Asian (n = 28)	28.5 ± 2.6	27.6 ± 2.5	29.2 ± 2.5	0.11

Numbers represent mean ± standard deviation. BSA: body surface area; EF: ejection fraction; FAC: fractional area change; GLS: global longitudinal strain; LA: left atrium; LV: left ventricle; RV: right ventricle; RVFWLS: right ventricular free wall longitudinal strain.

Among the 527 participants classified as healthy (50.2 years; 59% females), the mean LVGLS was 19.0% (95% CI: 14.3% to 23.8%), while the mean value of RVFWLS was 28.3% (95% CI: 22.3% to 34.3%). Females exhibited higher absolute values of both LVGLS and RVFWLS (Table 3). However, there were no differences in LVGLS and RVFWLS values across different age groups or in sex-stratified analysis (Figure 2). Table 4 presents the clinical and echocardiographic characteristics of this healthy subsample stratified by sex.

**Table 3:** – Reference values of longitudinal strain in the healthy subsample

	n	Mean ± SD	Limits of normal (95% CI)	p value
**LVGLS, %**				
Overall	527	19.0 ± 2.4	14 a 24	-
Male	215	18.3 ± 2.3	14 a 23	<0.01
Female	312	19.5 ± 2.3	15 a 24	
**RVFWLS, %**				
Overall	527	28.3 ± 3.1	22 a 34	-
Male	215	28.1 ± 3.1	22 a 34	0.07
Female	312	28.5 ± 3.0	23 a 34	

CI: confidence interval; LVGLS: left ventricle global longitudinal strain; RVFWLS: right ventricular free wall longitudinal strain; SD: standard deviation.

**Figure 2:** – Values of LVGLS and RVFWLS across ages in males and females in the healthy subsample. LVGLS: left ventricular global longitudinal strain; RVFWLS: right ventricular free wall longitudinal strain.

**Table 4:** – Baseline clinical and echocardiographic characteristics in the healthy subsample

	Overall (n = 527)	Male (n = 215)	Female (n = 312)	p value
**Age, years**	50.2 ± 8.1	49.6 ± 8.4	50.6 ± 7.9	0.13
**Self-identified skin color, n (%)**				
White	291 (55)	116 (53)	175 (56)	0.23
Black	75 (14)	25 (12)	50 (16)	0.15
Brown (pardo)	137 (26)	63 (29)	74 (23.7)	0.15
Indigenous	8 (2)	4 (2)	4 (1)	0.59
Asian	13 (2)	7 (3)	6 (2)	0.33
**Height, cm**	164.9 ± 9.3	172.9 ± 6.8	159.4 ± 6.4	<0.01
**BSA, m^2^**	1.76 ± 0.2	1.91 ± 0.17	1.67 ± 0.15	<0.01
**BMI, kg/m^2^**	25.7 ± 3.99	25.9 ± 3.76	25.5 ± 4.14	0.3
**Echocardiographic parameters**				
**LA diameter, cm**	3.4 ± 0.4	3.6 ± 0.4	3.3 ± 0.4	<0.01
**LA volume, mL**	47.5 ± 13	51.3 ± 13.7	45.2 ± 12.1	<0.01
**BSA-indexed LA volume, mL/m2**	26.8 ± 6.3	26.7 ± 6.4	26.9 ± 6.2	0.7
**LV diastolic diameter, cm**	4.4 ± 0.4	4.7 ± 0.4	4.3 ± 0.4	<0.01
**LV systolic diameter, cm**	2.8 ± 0.4	3.0 ± 0.4	2.7 ± 0.3	<0.01
**LV end-diastolic volume, mL**	89.9 ± 18.1	100 ± 17.7	83 ± 14.8	<0.01
**BSA-indexed LV end-diastolic volume, mL/m2**	51.7 ± 10.2	54.4 ± 10.9	49.9 ± 9.3	<0.01
**LV end-systolic volume, mL**	29.6 ± 8.5	33.4 ± 9.9	27.2 ± 6.4	<0.01
**BSA-indexed LV end-systolic volume, mL/m2**	17.2 ± 5.7	18.5 ± 6.9	16.3 ± 4.5	<0.01
**BSA-indexed LV mass, g/m^2^**	70.8 ± 14.5	77.4 ± 13.7	66.4 ± 13.4	<0.01
**LV mass/height2.7, g/m2.7**	32.4 ± 7.2	33.6 ± 6.8	31.6 ± 7.3	<0.01
**Relative wall thickness**	0.39 ± 0.05	0.39 ± 0.05	0.39 ± 0.06	0.5
**Mitral E to e’ ratio**	6.8 ± 1.6	6.5 ± 1.6	7.1 ± 1.6	<0.01
**Mean mitral annulus e’, cm/s**	10.8 ± 2.2	10.5 ± 2.1	10.9 ± 2.3	0.06
**RV basal diameter, cm**	3.5 ± 0.4	3.7 ± 0.3	3.3 ± 0.3	<0.01
**LVEF (Simpson method), %**	60.2 ± 6.1	59.4 ± 6.1	60.7 ± 6.0	0.01
**LVGLS, %**	19.0 ± 2.4	18.3 ± 2.3	19.5 ± 2.3	<0.01
White (n = 291)	18.9 ± 2.4	18.1 ± 2.2	19.5 ± 2.4	<0.01
Black (n = 75)	19.2 ± 1.8	19 ± 2.5	19.3 ± 1.8	0.53
Brown (pardo) (n = 137)	19 ± 2.5	18.2 ± 2.3	19.7 ± 2.4	<0.01
Indigenous (n = 8)	19 ± 2.2	19 ± 3.2	19 ± 1	0.99
Asian (n = 13)	19.2 ± 2.1	18.3 ± 2	20.2 ± 1.9	0.1
**RVFAC, %**	43.8 ± 5.7	42.8 ± 5.7	44.5 ± 5.6	<0.01
**RVFWLS, %**	28.3 ± 3.1	28.1 ± 3.1	28.5 ± 3.0	0.07
White (n = 291)	28.5 ± 3.1	27.9 ± 3.1	28.8 ± 3.1	0.01
Black (n = 75)	28.2 ± 2.9	28.2 ± 3	28.3 ± 2.9	0.95
Brown (pardo) (n = 137)	28 ± 3	28 ± 3.2	28 ± 2.7	0.9
Indigenous (n = 8)	29.3 ± 2.2	29.5 ± 2.5	29.2 ± 2.2	0.85
Asian (n = 13)	28 ± 2.4	27.8 ± 2.6	28.2 ± 2.4	0.77

Numbers represent mean ± standard deviation. BMI: body mass index; BSA: body surface area; EF: ejection fraction; FAC: fractional area change; GLS: global longitudinal strain; LA: left atrium; LV: left ventricle; RV: right ventricle; RVFWLS: right ventricular free wall longitudinal strain.

Abnormal LVGLS (< 14%) was detected in 3.8% of all participants, while abnormal RVFWLS (< 22%) was found in 1.6%. Supplementary Figure 1 shows the distribution of LVGLS and RVFWLS among the study population. Higher rates of abnormal LVGLS, but not RVFWLS, were observed in participants with hypertension and obesity (Figure 3; Supplementary Table 1).

**Figure 3:** – Distribution of abnormal LVGLS and RVFWLS among different clinical characteristics in the study population. LVGLS: left ventricular global longitudinal strain; RVFWLS: right ventricular free wall longitudinal strain.

In the tertile analysis, individuals in the worst LVGLS tertile group were predominantly males, and they had higher rates of hypertension, diabetes, and obesity, as well as higher cardiovascular risk scores. Additionally, these participants exhibited larger LV and RV dimensions, higher LV mass, increased relative wall thickness and higher LV concentric hypertrophy proportion. Moreover, worse LVGLS was associated with poorer LV and RV systolic parameters, as assessed by LVEF, RVFAC, and RVFWLS (Table 5). Regarding RVFWLS tertiles, participants with worse RVFWLS had higher body mass index (BMI), increased LV mass, and poorer LV and RV ventricular systolic parameters as assessed by LVEF, LVGLS, and RVFAC (Table 6). Figure 4 illustrates the factors correlated with abnormal LVGLS and RVFWLS. The primary findings of our study are presented in the Central Illustration.

**Table 5:** – Tertiles of left ventricle global longitudinal strain in the study population

	LVGLS			p value
	Better		Worse	
	←		→	
	Tertile 1 (n = 350) 26.73% to 19.71%	Tertile 2 (n = 349) 19.71% to 17.64%	Tertile 3 (n = 349) 17.63% to 7.12%	
				
Demographic parameters				
**Age, years**	51.9 ± 8.6	52.1 ± 8.9	51.9 ± 8.6	0.91
**Female, n (%)**	230 (65)	191 (54)	138 (39)	<0.01
**BMI, kg/m2**	25.9 ± 4	26.4 ± 4.2	27.4 ± 4.6	<0.01
**Hypertension, n (%)**	100 (28.5)	114 (32.6)	144 (41.2)	<0.01
**Diabetes, n (%)**	40 (11.4)	53 (15.1)	73 (20.9)	<0.01
**Current smoker, n (%)**	37 (10.5)	43 (12.3)	46 (13.2)	0.01
**Obesity, n (%)**	53 (15.1)	59 (16.9)	87 (24.9)	<0.01
**Overweight, n (%)**	142 (40.5)	146 (41.8)	158 (45.2)	0.42
**Chronic kidney disease, n (%)**	24 (6.8)	30 (8.5)	38 (10.8)	0.16
**Chronic obstructive pulmonary disease, n (%)**	3 (0.8)	7 (2)	5 (1.4)	0.44
**Stroke, n (%)**	4 (1.1)	3 (0.8)	3 (0.8)	0.9
**Heart failure, n (%)**	3 (0.8)	2 (0.5)	9 (2.5)	0.04
**Previous myocardial infarction, n (%)**	7 (2)	7 (2)	6 (1.7)	0.9
**ASCVD risk score, %**	1.95 [0.82 a 5.46]	2.7[1.1 a 6.85]	4 [1.4 a 9.3]	<0.01
**Echocardiographic parameters**				
**LA volume, mL**	47.8 ± 12.04	47.9 ± 13.4	48.3 ± 15.4	0.92
**BSA-indexed LA volume, mL/m2**	27.3 ± 6.3	26.7 ± 6.5	25.9 ± 7.3	0.02
**LV diastolic diameter, cm**	4.4 ± 0.4	4.4 ± 0.4	4.5 ± 0.5	<0.01
**LV mass, g**	126.8 ± 31.1	132.1 ± 35.5	147 ± 43.7	<0.01
**BSA-indexed LV mass, g/m2**	72.2 ± 14.7	73.5 ± 16.1	79.1 ± 19.3	<0.01
**LV mass/height2.7, g/m2.7**	33.5 ± 7.4	34 ± 8.3	36.6 ± 9.8	<0.01
**LV geometric patterns, n (%)**				
Concentric remodeling	98 (28)	95 (27.2)	113 (32.3)	0.27
Concentric hypertrophy	9 (2.6)	15 (4.3)	25 (7.2)	0.01
Eccentric hypertrophy	20 (5.7)	11 (3.1)	17 (4.9)	0.25
**Relative wall thickness**	0.39 ± 0.062	0.4 ± 0.067	0.42 ± 0.074	<0.01
**Mitral E to e’ ratio**	7.4 ± 1.8	7.1 ± 1.9	7.3 ± 1.9	0.3
**Mean mitral annulus e’, cm/s**	10.6 ± 2.3	10.3 ± 2.5	9.3 ± 2.3	0.32
**LVEF (Simpson method), %**	63.9 ± 5.1	59.8 ± 4.5	54.7 ± 5.4	<0.01
**RV basal diameter, cm**	3.5 ± 0.3	3.5 ± 0.4	3.5 ± 0.4	0.01
**RVFAC, %**	44.1 ± 5.5	43.8 ± 5.6	43.4 ± 5.7	0.21
**RVFWLS, %**	28.7 ± 3	28.1 ± 3.3	27.8 ± 3.2	<0.01

Numbers represent mean ± standard deviation for continuous variables with normal distribution, median [interquartile range] for continuous variables with abnormal distribution, and n (%) for categorical variables. Continuous variables were compared using a 2-sided t test with unequal variance for normally distributed data, the Wilcoxon signed rank test for abnormally distributed data, and chi-squared tests for categorical variables. ASCVD: atherosclerotic cardiovascular disease; BMI: body mass index; BSA: body surface area; EF: ejection fraction; FAC: fractional area change; GLS: global longitudinal strain; LA: left atrium; LV: left ventricle; RV: right ventricle; RVFWLS: right ventricular free wall longitudinal strain.

**Table 6:** – Tertiles of right ventricular free wall longitudinal strain in the study population

	RVFWLS			
	Better		Worse	p value
	←		→	
	Tertile 1 (n = 350) 26.73% to 19.71%	Tertile 2 (n = 349) 19.71% to 17.64%	Tertile 3 (n = 349) 17.63% to 7.12%	
Demographic parameters				
**Age, years**	53 ± 8.8	51.3 ± 8.2	51.7 ± 9	<0.01
**Female, n (%)**	197 (56)	192 (55)	170 (48)	0.1
**BMI, kg/m2**	26.1 ± 4	26.5 ± 4.7	27.1 ± 4.7	<0.01
**Hypertension, n (%)**	110 (31.4)	125 (35.8)	123 (35.2)	0.44
**Diabetes, n (%)**	54 (15.4)	56 (16)	56 (16)	0.96
**Current smoker, n (%)**	46 (13.1)	38 (10.9)	42 (12)	0.62
**Obesity, n (%)**	56 (16)	64 (18.3)	79 (22.6)	0.07
**Overweight, n (%)**	139 (39.7)	160 (45.8)	147 (42.1)	0.25
**Chronic kidney disease, n (%)**	27 (7.7)	28 (8)	37 (10.5)	0.33
**Chronic obstructive pulmonary disease, n (%)**	4 (1.1)	6 (1.7)	5 (1.4)	0.81
**Stroke, n (%)**	2 (0.6)	4 (1.1)	4 (1.1)	0.66
**Heart failure, n (%)**	4 (1.1)	7 (2)	3 (0.8)	0.38
**Previous myocardial infarction, n (%)**	4 (1.1)	8 (2.3)	8 (2.3)	0.43
**ASCVD risk score, %**	3.3 [1.1 a 7.6]	2.3 [1.1 a 6.1]	2.9 [1.1 a 6.9]	0.37
**Echocardiographic parameters**				
**LA volume, mL**	48.6 ± 14.3	47.5 ± 12.4	47.9 ± 14.1	0.58
**BSA-indexed LA volume, mL/m2**	27.2 ± 7.2	26.3 ± 6	26.4 ± 6.9	<0.01
**LV diastolic diameter, cm**	4.5 ± 0.4	4.5 ± 0.5	4.4 ± 0.5	0.64
**LV mass, g**	133.1 ± 37.7	137.1 ± 38.8	135.2 ± 37.3	<0.01
**BSA-indexed LV mass, g/m2**	74.3 ± 17	76 ± 17.5	74.3 ± 16.3	0.34
**LV mass/height2.7, g/m2.7**	34.2 ± 8.6	35.2 ± 8.6	34.7 ± 8.7	0.97
**LV geometric patterns, n (%)**				
Concentric remodeling	91 (26)	103 (29.5)	112 (32)	0.2
Concentric hypertrophy	17 (4.8)	18 (5.1)	14 (4)	0.75
Eccentric hypertrophy	16 (4.6)	19 (5.4)	13 (3.7)	0.55
**Relative wall thickness**	0.40 ± 0.067	0.41 ± 0.071	0.41 ± 0.066	0.23
**Mitral E to e’ ratio**	7.4 ± 1.9	7.2 ± 1.8	7.1 ± 1.9	0.21
**Mean mitral annulus e’, cm/s**	10.1 ± 2.4	10.2 ± 2.4	9.9 ± 2.4	0.86
**LVEF (Simpson method), %**	60.2 ± 6.2	59.5 ± 6.5	58.7 ± 6.1	<0.01
**LVGLS, %**	19 ± 2.5	18.6 ± 2.6	18.3 ± 2.5	<0.01
**RV basal diameter, mm**	35.2 ± 3.7	34.9 ± 4.3	34.8 ± 3.7	0.3
**RVFAC, %**	45.6 ± 5.4	44 ± 5.3	41.8 ± 5.6	<0.01

Numbers represent mean ± standard deviation for continuous variables with normal distribution, median [interquartile range] for continuous variables with abnormal distribution, and n (%) for categorical variables. Continuous variables were compared using a 2-sided t test with unequal variance for normally distributed data, the Wilcoxon signed rank test for abnormally distributed data, and chi-squared tests for categorical variables. ASCVD: atherosclerotic cardiovascular disease; BMI: body mass index; BSA: body surface area; EF: ejection fraction; FAC: fractional area change; GLS: global longitudinal strain; LA: left atrium; LV: left ventricle; RV: right ventricle; RVFWLS: right ventricular free wall longitudinal strain

**Figure 4:** – Clinical and echocardiographic characteristics associated with worse LVGLS and RVFWLS in the study population. BMI: body mass index; DM: diabetes mellitus; HTN: hypertension; LV: left ventricle; LVEF: left ventricular ejection fraction; LVGLS: left ventricular global longitudinal strain; RV: right ventricle; RVFAC: right ventricular fractional area change; RVFWLS: right ventricular free wall longitudinal strain.

Regression analysis revealed a significant association between LVEF and LVGLS (r = −0.66, p < 0.01), as well as between RVFWLS and RVFAC (r = −0.31, p < 0.01) in the total population. Similar associations were observed in the healthy population (r = −0.65, p < 0.01; r = −0.32, p < 0.01, respectively), as illustrated in Supplementary Figure 2.

## Discussion

To the best of our knowledge, this is the largest Brazilian cohort used to estimate reference values for LVGLS and RVFWLS in a multiethnic adult population. Our findings indicate significant differences in LVGLS and RVFWLS according to sex, but not age. We found that individuals with reduced absolute values of LVGLS were predominantly males, with higher rates of hypertension, diabetes, and obesity; they exhibited larger ventricular dimensions, worse LV concentric hypertrophy, as well as worse LVEF and RV systolic parameters. Additionally, participants with reduced absolute RVFWLS values had higher BMI and LV mass, along with worse LVEF and RVFAC. These findings suggest a complex interplay between demographic, clinical, and echocardiographic factors influencing LVGLS and RVFWLS.

The average reference value of LVGLS reported in our study was similar to other studies. A published meta-analysis, which included more than 2,500 participants from 24 studies, reported a normal LVGLS value of 19.7% (95% CI: 18.9% to 20.4%),^15^ and a recent smaller cross-sectional study, including healthy Brazilian participants (n = 77), presented 19% ± 2% as the reference value.^16^ Our mean absolute reference RVFWLS value was similar to some studies,8 but higher compared to others. In a Danish prospective cohort study of patients without cardiovascular diseases or risk factors, the mean value of RVFWLS was 26.7% ± 5.2%.^17^ Nyberg et al. published reference values for GLS in a Norwegian cohort,^18^ showing a mean RVFWLS value of 25.9% (95% CI: 17.4% to 34.5%). Another study in healthy individuals conducted in India demonstrated a mean RVFWLS value of 23.6% ± 3.8%.19 Potential explanations for the disparity in these results may be attributed to ethnicity-related differences and the utilization of distinct software for RV strain analysis. Our data suggest that reference values for these echocardiographic measures can vary significantly across populations, and the application of global values may be inaccurate.

There is increasing evidence about the impact of sex and age on LVGLS,20 even when this parameter was obtained using other methods such as cardiac magnetic resonance.21 We found a 1.3% higher LVGLS absolute value in females compared to males, which is consistent with previously reported values in a general population without cardiovascular disease or traditional risk factors.16 Moreover, we reaffirmed the effect of sex on LV myocardial deformation expressed by a lower prevalence of females in the lowest tertile of LVGLS. Regarding RVFWLS, we observed a tendency towards higher absolute values among females within our sample. Sex differences in systolic function may be explained by structural differences observed in females, characterized by smaller cavities. However, neurohormonal and other biological factors can also influence this process, particularly among females under 60 years of age.22 The effects of age on myocardial deformation remain a topic of controversy. In the cohort study conducted by Sengupta et al.,19 involving healthy volunteers, no age-related disparity in LVGLS was observed. Furthermore, as per the findings of Espersen et al.,17 in their cohort study, age did not have an independent association with RVFWLS in multivariable linear regression analysis. Within our cohort, age did not demonstrate a significant association with GLS. Nevertheless, it is worth noting that the relatively narrow age range in our sample might have limited our ability to detect differences in GLS across various age groups.

Our study found a significantly higher proportion of abnormal LVGLS values in participants with cardiovascular disease risk factors compared to those without such risk factors. In contrast, there was no statistically significant difference in the proportion of abnormal RVFWLS values between participants with and without cardiovascular disease risk factors. Furthermore, worse LVGLS was associated with cardiovascular risk factors, such as hypertension, diabetes, and obesity, similar to findings reported in other studies.^23^ In hypertension, a reduction in LVGLS values was described even at the early stages of the disease spectrum, with differences detected between prehypertension and stage I hypertension (LVGLS 17.5% ± 2.5% versus 18.2% ± 2.4%, p = 0.03), as shown in a Brazilian study.^24^ Individuals with diabetes also presented worse LVGLS values,^2^5 and a recent study showed that treatment with hypoglycemic drugs (sodium–glucose transporter 2 inhibitors) improved this parameter, even after a short follow-up period.^26^ Additionally, obesity was linked with subclinical reduction in myocardial deformation measured by LV and RV strain, even in individuals without any cardiovascular disease, and the degree of BMI elevation was associated with an incremental risk of subclinical myocardial dysfunction.^27^ Other cardiovascular risk factors, besides BMI, were not associated with worse RVFWLS. RV functional impairment is most likely multifactorial, attributable both to conditions leading to RV pressure overload due to elevated pulmonary pressure and to volume overload resulting from LV dysfunction, culminating in biventricular failure.^28^ This premise suggests that RV strain may hold greater clinical relevance in disease-specific contexts predominantly involving right-side chambers, rather than demonstrating utility within the general population with low cardiovascular risk.

Considering echocardiographic findings, our study revealed that worse LVGLS and RVFWLS were associated with LV hypertrophy, as indicated by increased LV mass, reinforcing the hypothesis that LV hypertrophy may induce a progressive alteration in subendocardial and subepicardial myocardial fibers, initially characterized by attenuation of longitudinal strain.^29^ Furthermore, larger ventricular dimensions were associated with worse LVGLS, suggesting a greater susceptibility of the endocardial layer to hemodynamic insults. Yoshida et al. propose several mechanisms to explain the independent association between GLS and LA and RV dysfunction. One proposed mechanism is impaired coronary microcirculation, which can negatively affect both ventricular and atrial function. Additionally, anatomical interactions might explain these relationships. LV function modulates LA reservoir function through the systolic downward motion of the LV base. Regarding ventricular interdependence, the LV and RV share myofibers that encircle both ventricles.^30^ The association between LV and RV strain and other traditional parameters of LV and RV function (LVEF and RVFAC) highlights the ventricular interdependence and the strong connection between LV and RV chambers.^17^

### Limitations

Some limitations of this analysis should be noted. The echocardiographic images were acquired between 2008 and 2010, following the guidelines in effect at that time. Strain analysis was not initially anticipated or incorporated into the original protocol. As a result, our analysis of LVGLS was based on offline assessment of apical 4- and 2-chamber views. Consequently, we used the 12-segment model for LVGLS analysis, which was commonly applied in other large-scale populations.^31,32^ Furthermore, our strain findings should be interpreted considering that images were analyzed using vendor-independent software, and the reported normal values may not be directly transferable to measures performed using vendor-specific software. Although 3-dimensional speckle-tracking echocardiography has the potential for a more accurate assessment of myocardial function,^33^ the protocol of the ELSA-Brasil required only 2-dimensional images. Another limitation of the present study is the low inter-observer ICC for both LVGLS and RVFWLS measurements, similar to previous studies that can reflect the learning curve of strain analysis, especially for RV strain. Inter-observer variability, particularly for LVGLS, has been documented in the literature and may result from differences in observer experience, image quality, and software usage. Additionally, the study is subject to potential selection bias due to the use of a cohort of civil servants from universities and research institutions, which may not be fully representative of the general Brazilian population. Finally, our narrow age range can limit the generalizability of these findings to other age groups.

## Conclusion

Our study within the large, middle-aged, Brazilian, multiethnic population from the ELSA-Brasil cohort provides valuable insights into the reference values of GLS for both left and right ventricles. Additionally, we were able to demonstrate that reduced LVGLS and RVFWLS were associated with cardiovascular comorbidities, cardiac structure, and function, using frequently employed echocardiographic parameters, especially LVGLS.
